# Prevalence and Hospital Management of Amphotericin B Deoxycholate-Related Toxicities during Treatment of HIV-Associated Cryptococcal Meningitis in South Africa

**DOI:** 10.1371/journal.pntd.0004865

**Published:** 2016-07-28

**Authors:** Susan Meiring, Melony Fortuin-de Smidt, Ranmini Kularatne, Halima Dawood, Nelesh P. Govender, GERMS-SA

**Affiliations:** 1 National Institute for Communicable Diseases (NICD), a Division of the National Health Laboratory Service (NHLS), Johannesburg, South Africa; 2 Faculty of Health Sciences, University of the Witwatersrand, Johannesburg, South Africa; 3 National Health Laboratory Service, Johannesburg, South Africa; 4 Pietermaritzburg Hospital Complex, Pietermaritzburg and University of KwaZulu-Natal, Durban, South Africa; 5 Faculty of Health Sciences, University of Cape Town, Cape Town, South Africa; University of Tennessee, UNITED STATES

## Abstract

**Background:**

We aimed to establish the prevalence of amphotericin B deoxycholate (AmBd)-related toxicities among South African patients with cryptococcosis and determine adherence to international recommendations to prevent, monitor and manage AmBd-related toxicities.

**Methods:**

Clinical data were collected from cases of laboratory-confirmed cryptococcosis at 25 hospitals, October 2012 –February 2013. Anemia was defined as hemoglobin (Hb) concentration <10 g/dl, hypokalemia as serum potassium (K) <3.4 mEq/L and nephrotoxicity as an increase in serum creatinine (Cr) to >1.1 times the upper limit of normal. To determine adherence to toxicity prevention recommendations, we documented whether baseline Hb, K and Cr tests were performed, whether pre-emptive hydration and IV potassium chloride (KCl) was administered prior to 80% and 60% of AmBd doses and whether daily oral KCl supplementation was given ≥60% of the time. To determine adherence to monitoring recommendations, we ascertained whether a daily fluid chart was completed, Hb was monitored weekly and K or Cr were monitored bi-weekly.

**Results:**

Of 846 patients, clinical data were available for 76% (642/846), 82% (524/642) of whom received AmBd. Sixty-four per cent (n = 333) had documented baseline laboratory tests, 40% (n = 211) were given pre-emptive hydration and 14% (n = 72) and 19% (n = 101) received intravenous and oral KCl. While on AmBd, 88% (n = 452) had fluid monitoring; 27% (n = 142), 45% (n = 235) and 44% (n = 232) had Hb, K and Cr levels monitored. Toxicities developed frequently during treatment: anemia, 16% (86/524); hypokalemia, 43% (226/524) and nephrotoxicity, 32% (169/524).

**Conclusion:**

AmBd-related toxicities occurred frequently but were potentially preventable with adequate monitoring, supplemental fluid and electrolyte therapies.

## Author Summary

Amphotericin B deoxycholate (AmBd)-based induction therapy is recommended for treatment of HIV-associated cryptococcosis in South Africa. We reviewed 846 patient records to determine adherence to World Health Organization (WHO) recommendations to prevent, monitor and manage AmBd-related toxicities and determine the prevalence of such toxicities.

## Introduction

*Cryptococcus neoformans* is a leading cause of meningitis among severely immunocompromised HIV-infected adults and even with amphotericin B-based therapy, the mortality is high [1–3]. Advanced HIV disease at diagnosis (CD4+ T-lymphocyte (CD4) count <200 cells/μl), high cerebrospinal fluid (CSF) fungal burden, sub-optimal management of cryptococcal meningitis (CM), delayed initiation of antiretroviral treatment (ART) and the presence of co-morbid conditions such as tuberculosis all contribute to the high case-fatality ratio (CFR) [4–6].

Amphotericin B deoxycholate (AmBd), in combination with fluconazole, is recommended as first-line induction therapy for the treatment of CM in South Africa (AmBd 1 mg/kg/day plus oral fluconazole 800 mg daily). Since publication of recommendations by the South African HIV Clinicians’ Society (SAHIVCS) in 2007, the proportion of patients treated with AmBd-based therapy for CM has increased from 30% to 80% [7–10]. Despite this increase, there has been no associated decrease in the in-hospital CFR which has remained between 31% and 34% since 2007[11, 12].

AmBd is rapidly fungicidal against *Cryptococcus*; however, the antifungal agent has several adverse effects due to its binding with cholesterol in human cell membranes [13, 14]. The most common adverse events include nephrotoxicity, hypokalemia, hypomagnesemia, anemia, febrile reactions and chemical phlebitis. An observational study from Kenya reported nephrotoxicity in 59%, hypokalemia in 93%, and hypomagnesaemia in 80% of 70 patients with CM treated with AmBd[15]. In clinical trial settings with standardized pre-hydration and electrolyte supplementation regimens, 10% of patients still develop nephrotoxicity and 6% develop hypokalemia [16–18]. A recent study reported improved 30-day survival following proactive fluid and electrolyte management of patients with CM treated with AmBd.[19] Following recommendations by the WHO, South African guidelines were modified to include a minimum package of interventions to prevent, monitor and treat AmBd-related toxicities [7, 20].

Currently, there are few real-world data on the prevalence of AmBd toxicities and the use of interventions to prevent, monitor and treat these toxicities in the context of HIV-associated CM. In this observational study conducted at 25 sentinel South African hospitals, we aimed to establish the prevalence of AmBd-related toxicities among patients with CM, to determine physician awareness of the recommendations and to determine if these recommendations were being followed.

## Methods

### Study setting and design

This prospective, observational cross-sectional study was nested within national, laboratory-based, population-based surveillance for cryptococcosis in South Africa, conducted by the National Institute for Communicable Diseases (GERMS-SA)[21]. A case of cryptococcosis was defined as a person who was seen at a South African healthcare facility with a positive CSF India ink test, a positive cryptococcal antigen test (on CSF or serum) or culture of *Cryptococcus* from any anatomical specimen. If a subsequent specimen from the same person tested positive for any of the aforementioned tests >30 days after the first positive specimen, this was considered as a case of recurrent disease and included in the study. At 25 enhanced surveillance (ES) sites in nine provinces, clinical data (such as HIV infection status, CD4 count, ART use, mental status at diagnosis [assessed by using the Glasgow Coma Scale (GCS) score], antifungal treatment and in-hospital outcome) were collected through medical record review and/or interview. The ES sites are large hospitals in urban areas; 11 of the 25 facilities were categorized as academic hospitals (i.e. directly linked to a university medical school and conducted training of medical students and specialists). From October 2012 through February 2013, additional laboratory and clinical data were collected from adults who were prescribed AmBd in hospital and gave informed consent for both surveillance and this nested study.

### Study definitions

#### AmBd-related toxicities

Patients with anemia, hypokalemia or nephrotoxicity that was diagnosed prior to initiation of AmBd were excluded from prevalence calculations. Toxicities were determined according to the Division of AIDS grading criteria[22]. Anemia was defined as a hemoglobin (Hb) <10 g/dL, hypokalemia was defined as a serum potassium (K) <3.4 mEq/L and nephrotoxicity was defined as a serum creatinine (Cr) level that increased to >1.1 times the upper limit of normal (1.1 mg/dL). Toxicities were reported as potentially life-threatening (grade 4 of the severity of adverse events grading for laboratory tests) if Hb was <6.5 g/dL, serum K was <2.0 mEq/L and serum Cr increased to ≥3.5 times the upper limit of normal (1.1 mg/dL)[22]. No data were collected on thrombophlebitis.

#### Adherence to recommendations for prevention of AmBd-related toxicities

Four laboratory or clinical parameters were reviewed: (1) baseline Hb, K and Cr tests performed in the week prior to starting AmBd; (2) intravenous (IV) pre-emptive hydration with 1L of 0.9% saline administered prior to each AmBd dose; (3) 1 ampule of IV potassium chloride (KCl) administered prior to each AmBd dose and (4) oral KCl supplementation administered daily whilst the patients were receiving AmBd. For each parameter, optimal adherence was arbitrarily defined as: (1) all baseline tests were performed in the week prior to starting AmBd, (2) 1L of 0.9% saline was administered prior to at least 80% of AmBd doses, (3) IV KCl supplementation was administered prior to at least 60% of AmBd doses and (4) oral KCl was administered for at least 60% of the days during which the patient was on AmBd.

#### Adherence to recommendations for monitoring for AmBd-related toxicities

Four parameters were reviewed and while patients were on AmBd treatment, optimal adherence was defined as: (1) a completed daily fluid input/output chart; (2) weekly Hb tests; (3) biweekly serum K and (4) biweekly serum Cr tests.

### Physician interviews

From October 2012 through June 2013, we interviewed physicians who were currently involved in the daily management of patients with cryptococcosis at the 25 ES sites. We approached at least 4 physicians at each of the ES sites to participate in the study. A standardized study questionnaire was used to determine the knowledge and behavior of physicians with regard to prevention and monitoring of AmBd toxicities and the general in-hospital management of cryptococcosis.

### Statistical analysis

Data analysis was performed using Stata version 11.2 (StataCorp, College Station, Texas). Univariate and multivariable analyses were conducted to determine the factors that were associated with in-hospital outcome. The logistic regression model included ES site category [academic vs. non-academic], sex, CD4 count, ART use, mental status at diagnosis and whether or not recommendations to prevent and monitor AmBd-related toxicities were followed optimally. P values <0.05 were considered significant.

### Ethics

Approval to conduct cryptococcal disease surveillance was obtained from the following university-affiliated Human Research Ethics Committees: University of the Witwatersrand (WITS) (M081117); University of Cape Town (UCT) (115/2009); Stellenbosch University (SU) (N04/01/001) and University of KwaZulu-Natal (UKZN) (BF130/11). Approval for the nested study was obtained from SU and UKZN as amendments to the cryptococcal disease surveillance protocol and as a new study with unique references from WITS (M120845) and UCT (482/2012). Written informed consent was obtained from all interviewed patients and clinicians.

## Results

### Cases of cryptococcosis at ES sites

**Fig 1:**
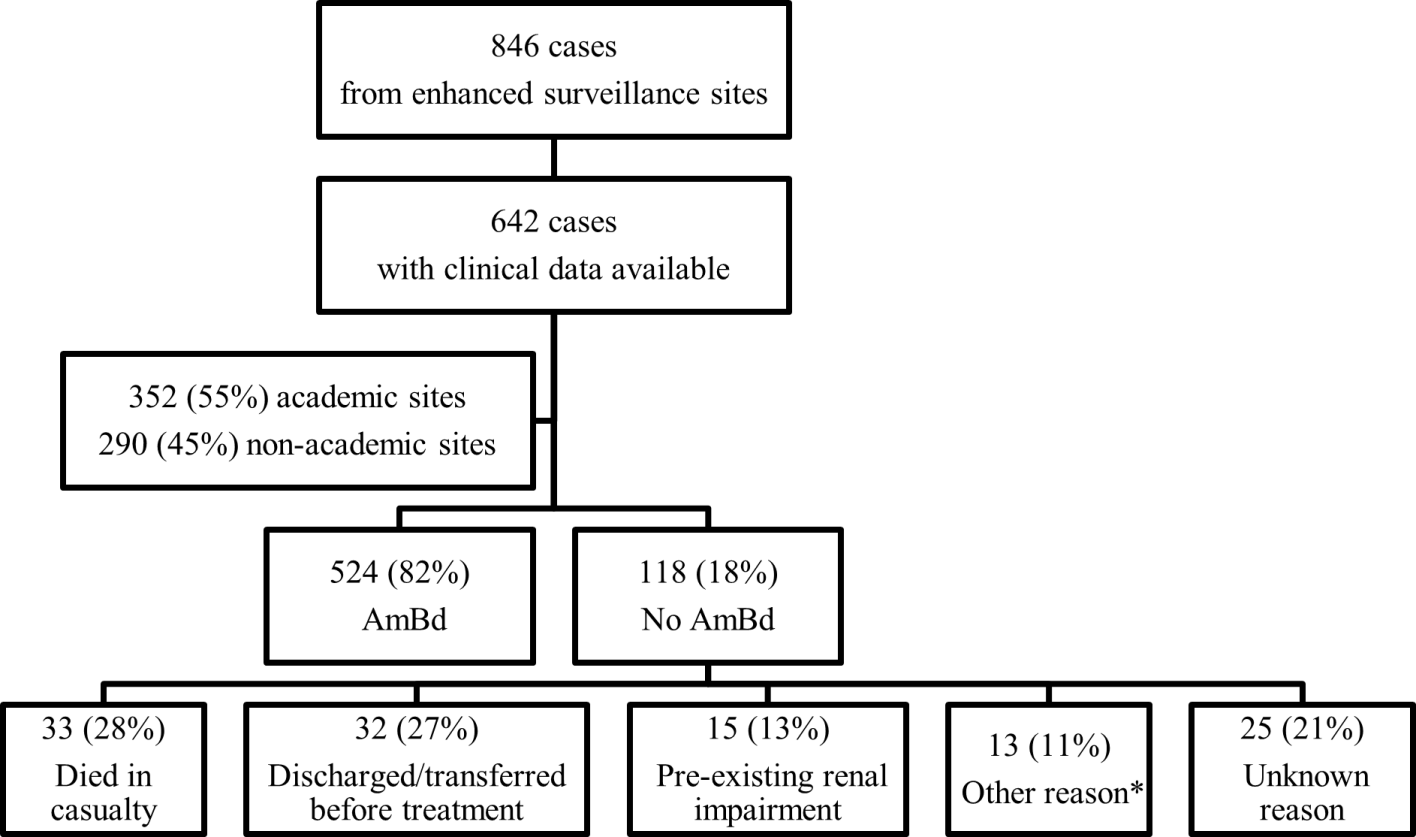
Enrolment of patients with cryptococcosis from 25 enhanced surveillance sites into the nested cross-sectional study, October 2012 through February 2013. Footnote: AmBd: amphotericin B deoxycholate. *Other reason: 10 (8%) no antifungal therapy was given; 3 (3%) AmBd was out of stock

During the five-month study period, of 846 unique episodes of cryptococcosis, clinical data were available for 642 (76%). (Fig 1) There were no significant differences in ES site category, sex or age for patients with available data compared to those with missing data. Of those with available data, 45% were female (288/640, sex unknown for 2 cases); all patients were HIV-infected; and 78% (351/452, CD4 count not done for 190 cases) had a CD4 count <100 cells/μl at diagnosis. Thirty-seven per cent (240/642) were on ART at diagnosis. Eighty-two per cent (524/642) of the patients were treated with AmBd with no significant difference by ES site category. (Table 1) Of 524 patients treated with AmBd, the median number of days on treatment was 11 (interquartile range (IQR), 6–14 days). The CFR for those on AmBd was 25% (134/524). Overall in-hospital CFR was 29% (187/642) (academic: 29% [103/352] vs. non-academic: 29% [84/290], p = 0.935).

**Table 1:** Number of patients with cryptococcosis and number of physicians interviewed by enhanced surveillance site (hospital) category and South African province.

Hospital category and province	Cryptococcal disease surveillance study				Interviews with physicians treating cryptococcal disease	
	Patients included in the study	Patients not treated with amphotericin B	Patients treated with amphotericin B	Hospitals included	Physicians interviewed	Hospitals included
**Academic hospitals (sub-total)**	**352**	**66**	**286**	**11**	**21**	**8**
-Eastern Cape	20	7	13	1	2	1
-Free State	1	0	1	1	0	0
-Gauteng	288	48	240	5	12	4
-KwaZulu-Natal	10	3	7	2	3	1
-Western Cape	33	8	25	2	4	2
**Non-academic hospitals (sub-total)**	**290**	**52**	**238**	**14**	**21**	**12**
-Eastern Cape	35	7	28	1	0	0
-Free State	20	4	16	1	2	1
-Gauteng	35	5	30	2	0	1
-KwaZulu-Natal	150	29	121	4	5	4
-Limpopo	9	0	9	2	6	2
-Mpumalanga	20	4	16	1	1	1
-Northern Cape	11	2	9	1	2	1
-North West	9	1	8	1	3	1
-Western Cape	1	0	1	1	2	1
**Total**	**642**	**118**	**524**	**25**	**42**	**20**

### Prevalence of AmBd-related toxicities

Prior to receiving AmBd therapy, there were 91 patients with pre-existing anemia, 101 with hypokalemia and 42 with renal dysfunction. During AmBd therapy, an additional 12% of patients (52/433) developed anemia, 39% (163/423) developed hypokalemia and 30% (144/482) developed nephrotoxicity. Of those who were optimally monitored for AmBd-related toxicities (and including those with pre-existing anemia, hypokalemia and nephrotoxicity), 54% (77/142) developed anemia, 71% (167/235) developed hypokalemia and 55% (127/232) developed nephrotoxicity (Table 2). Among those optimally monitored, the proportion of patients who developed toxicities was similar at academic vs. non-academic ES sites (p = 0.363 for anemia, p = 0.717 for hypokalemia and p = 0.101 for nephrotoxicity) and did not differ significantly if those with pre-existing anemia, hypokalemia and nephrotoxicity were excluded. Most cases of new-onset nephrotoxicity (57%; 82/144) developed during the second week of AmBd therapy. In most cases, the severity of complications was mild to moderate. Among those optimally monitored, potentially life-threatening (grade 4) toxicities occurred among 17% (13/77) of patients with anemia, 5% (8/167) of patients with hypokalemia and 6% (7/127) of patients with nephrotoxicity. Severe (grade 3) nephrotoxicity and anemia was significantly higher among patients treated with AmBd who had pre-existing nephrotoxicity and anemia (57% vs. 25% for grade 3 nephrotoxicity, p = 0.03; and 22% vs. 7% for grade 3 anemia, p = 0.03) (S1 Table).

**Table 2:** Comparison of patient outcome amongst those optimally monitored for nephrotoxicity, hypokalemia and anemia whilst receiving amphotericin B deoxycholate therapy for cryptococcosis.

**Toxicity**	**Died in hospital**		**Survived to discharge**		
	**No.**	**%**	**No.**	**%**	**p-value**
**Serum creatinine optimally monitored (n = 232)**					
- Nephrotoxicity present	27	21	100	79	0.8
- Nephrotoxicity absent	21	20	84	80	
**Serum potassium optimally monitored (n = 235)**					
- Hypokalemia present	36	22	131	78	0.5
- Hypokalemia absent	12	18	56	82	
**Hemoglobin optimally monitored (n = 142)**					
- Anemia present	21	27	56	73	0.9
- Anemia absent	17	26	48	74	

Serum creatinine (Cr) or serum potassium (K) optimally monitored: K or Cr measured biweekly whilst on AmBd therapy

Hemoglobin optimally monitored: hemoglobin measured weekly whilst on AmBd therapy

Nephrotoxicity: Serum creatinine >1.1 times upper limit of normal

Hypokalemia: Serum potassium <3.4 mEq/L

Anemia: Hemoglobin (Hb) concentration <10 g/dl

### Adherence to recommendations for prevention of AmBd-related toxicities

While just under two-thirds of cases (64%, 333/524) had Hb, K and Cr tests performed at baseline, optimal pre-emptive hydration was administered to 40% (211/524), optimal oral KCl supplementation to 19% (101/524) and optimal IV KCl supplementation to 14% (72/524) of patients. (Table 3) Patients treated with AmBd at academic ES sites were more likely to receive optimal IV KCl supplementation than at non-academic sites (20%, 57/286 vs. 6%, 15/238, p<0.001).

**Table 3:** Adherence to recommendations for prevention and monitoring of amphotericin B deoxycholate-related toxicities among patients with cryptococcosis (n = 524).

**Parameter**	**Followed/ monitored at any time**		**Followed/monitored optimally**	
	**n**	**%**	**n**	**%**
**Preventive parameters:**				
Baseline blood tests	.	.	333	64
- Baseline hemoglobin checked	348	66	.	.
- Baseline potassium checked	376	72	.	.
- Baseline creatinine checked	376	72	.	.
Pre-emptive hydration given	478	91	211	40
Intravenous potassium chloride given	241	46	72	14
Oral potassium chloride given	150	29	101	19
**Monitoring parameters:**				
Daily fluid input/output monitoring	.	.	452	88
Hemoglobin	172	33	142	27
Potassium (serum)	357	68	235	45
Creatinine (serum)	358	68	232	44

Optimal adherence to baseline blood test recommendations: hemoglobin, serum potassium and serum creatinine checked in the week preceding amphotericin B deoxycholate (AmBd) therapy

Pre-emptive hydration: 1L 0.9% saline administered prior to each dose of AmBd (optimal = pre-emptive hydration given for ≥80% of doses)

Intravenous potassium chloride (KCl): 1 ampoule (20 mEq) of KCl added to 1L normal saline prior to each dose of AmBd (optimal = given for ≥60% of doses)

Oral KCl: any tablets containing KCl administered daily whilst on AmBd (optimal = given for ≥60% of doses)

Optimal hemoglobin monitoring: hemoglobin measured weekly whilst on AmBd therapy

Optimal potassium (K) or creatinine (Cr) monitoring: K or Cr measured biweekly whilst on AmBd therapy.

### Adherence to recommendations for monitoring for AmBd-related toxicities

The majority of patients had daily fluid monitoring (86%, 452/524). However, only 45% (235/524) and 44% (232/524) of patients had serum K and Cr levels checked bi-weekly, whilst less than one third of patients had weekly monitoring of Hb levels. (Table 3) Patients at academic ES sites were significantly more likely to have all four aforementioned parameters monitored than those at non-academic ES sites (p<0.001). (S2 Table)

### Case-fatality ratio

On multivariable analysis, only a GCS score of 15 and optimal pre-emptive hydration during AmBd treatment was significantly associated with in-hospital survival. (Table 4) Among those optimally monitored for AmBd-related toxicities, there was also no significant difference in outcome between those who developed nephrotoxicity, hypokalemia or anemia (whether or not the patients with baseline anemia, hypokalemia or nephrotoxicity were included in the analysis) (Table 2). In addition, there was no difference in outcome by ES site category, sex, CD4 count, ART use, pre-existing anemia, pre-existing hypokalemia or pre-existing renal impairment. The exact cause of death for the 25% who died after receiving AmBd treatment was not determined.

**Table 4:** Univariate and multivariable analysis of factors associated with in-hospital outcome among patients treated with amphotericin B deoxycholate (n = 524).

**Parameter**	**Died in hospital**		**Survived to discharge**		**Univariate analysis**		**Multivariable analysis**	
	**n**	**%**	**n**	**%**	**Unadjusted Odds Ratio (95% CI)**	**p-value**	**Adjusted Odds Ratio (95% CI)**	**p-value**
Mental status*								
- GCS score <15	65	39	102	61	Reference		Reference	
- GCS score = 15	58	18	258	82	0.35 (0.23–0.54)	<0.001	0.38 (0.24–0.58)	<0.001
**Initial opening CSF pressure**								
- Unmeasured	114	26	322	74	Reference			
- Measured	17	20	66	80	0.73 (0.41–1.29)	0.278		
**Baseline laboratory testing (hemoglobin, potassium, creatinine)**								
- Suboptimal	49	26	142	74	Reference			
- Optimal	84	25	249	75	0.98 (0.65–1.47)	0.913		
**Pre-emptive hydration**								
- Suboptimal	106	34	207	66	Reference		Reference	
- Optimal	27	13	184	87	0.29 (0.18–0.46)	<0.001	0.34 (0.20–0.58)	<0.001
**Intravenous KCl administration**								
- Suboptimal	123	27	329	73	Reference		Reference	
- Optimal	10	14	62	87	0.43 (0.21–0.87)	0.018	0.72 (0.30–1.73)	0.465
**Oral KCl administration**								
- Suboptimal	117	28	306	72	Reference		Reference	
- Optimal	16	16	85	84	0.49 (0.28–0.87)	0.016	0.70 (0.38–1.29)	0.255
**Monitoring of K**								
- Suboptimal	85	29	204	71	Reference		Reference	
- Optimal	48	20	187	80	0.62 (0.41–0.92)	0.019	0.57 (0.10–3.27)	0.526
**Monitoring of Cr**								
- Suboptimal	85	29	207	71	Reference		Reference	
- Optimal	48	21	184	79	0.64 (0.42–0.95)	0.028	1.46 (0.25–8.46)	0.676
**Monitoring of Hb**								
- Suboptimal	95	25	287	75	Reference			
- Optimal	38	27	104	73	1.10 (0.71–1.71)	0.658		

*Mental status was missing for 41 patients

Optimal adherence to baseline blood test recommendations: hemoglobin, serum potassium and serum creatinine checked in the week preceding amphotericin B deoxycholate (AmBd) therapy

Pre-emptive hydration: 1L 0.9% saline administered prior to each dose of AmBd (optimal = pre-emptive hydration given for ≥80% of doses)

Intravenous potassium chloride (KCl): 1 ampoule (20 mEq) of KCl added to 1L normal saline prior to each dose of AmBd (optimal = given for ≥60% of doses)

Oral KCl: any tablets containing KCl administered daily whilst on AmBd (optimal = given for ≥60% of doses)

Optimal hemoglobin monitoring: hemoglobin measured weekly whilst on AmBd therapy

Optimal potassium (K) or creatinine (Cr) monitoring: K or Cr measured biweekly whilst on AmBd therapy.

### Physician knowledge and behavior

Forty-two physicians, ranging in experience from interns to certified specialists, were interviewed at 20 of the 25 ES sites (this included at least one hospital from each of South Africa’s 9 provinces). (Table 1) Half the physicians were from academic sites and the other half from non-academic sites. At academic sites, most interviews were conducted with specialist trainees (registrars; 38% [8/21]) while non-specialist medical officers were most often interviewed at non-academic sites (48%, 10/21). There was no significant difference in responses between physicians at academic vs. non-academic sites. While 67% (28/42) reported that they were aware of any guideline for the management of cryptococcal disease, only 26% (11/42) were aware of the WHO Rapid Advice guidelines published a year earlier. The majority (38/42, 95%) indicated that they used AmBd as first-line management and 93% (39/42) were aware of potential toxicities associated with its use. When asked about interventions to prevent AmBd toxicities, 79% (33/42) reported prescribing pre-emptive hydration, 71% (30/42) said that they checked baseline Hb levels, 90% (40/42) reported checking baseline serum Cr and K and 71% (30/42) said that they prescribed routine electrolyte supplementation. However, when asked to specify the type of electrolyte supplementation, 40% (17/42) said that they prescribed oral KCl daily and only 5% (2/42) prescribed IV KCl. Almost all (40/42) claimed to monitor serum Cr and K levels during AmBd treatment, though only 60% (24/40) ordered these tests twice a week.

## Discussion

While the vast majority of South African patients with cryptococcosis in this urban, hospital-based observational study were treated with AmBd, the overall in-hospital CFR was high. A GCS score of 15 and pre-emptive hydration during AmBd treatment was associated with in-hospital survival. AmBd-related toxicities developed frequently during treatment, including half of patients who developed nephrotoxicity, but most toxicities were graded mild to moderate. Almost two-thirds of patients had baseline laboratory tests performed but less than half received pre-emptive hydration and less than a fifth were prescribed pre-emptive potassium supplementation. Fluid monitoring was well implemented though less than half of patients had appropriate monitoring laboratory tests ordered. These findings contrast starkly with physician perceptions of management of AmBd toxicities. While most physicians prescribed AmBd as first-line therapy and were aware of the potential toxicities associated with its use, less than a third were aware of published recommendations to mitigate these toxicities.

Eighty per cent of patients admitted with cryptococcosis received AmBd induction therapy in 2012–2013. This is a substantial improvement since 2008 when only 30% of patients received AmBd[11, 12]. Possible reasons for increased use of AmBd include a reduction in the cost of AmBd, inclusion in the South African hospital-level essential medicines list, improved availability in hospital pharmacies and publication of local guidelines on cryptococcal disease management [7, 8, 23]. However, many patients still do not receive adequate induction-phase treatment due to the additional nursing time required to manage an IV infusion, the need to hospitalize patients while receiving AmBd, the perception that AmBd-related toxicities are unmanageable and the need for access to laboratory services to monitor for AmBd-related toxicities.

Among patients on AmBd therapy, those who were given a liter of normal saline intravenously prior to each dose of AmBd were 70% more likely to survive than those who did not receive optimal pre-emptive hydration, even when controlling for altered mental status which is a major contributor to acute mortality [3, 4]. This intervention may improve survival by decreasing AmBd-related nephrotoxicity and electrolyte abnormalities [16, 19, 24]. Unfortunately, we could not test this hypothesis by including nephrotoxicity, hypokalemia and anemia into our multivariate model as monitoring for these laboratory-diagnosed toxicities was very poor. We did not show any difference in in-hospital mortality among those who were both optimally monitored for AmBd-related toxicities and developed toxicities. This is not surprising since these patients were potentially already receiving superior care than those with no or suboptimal monitoring for AmBd-related toxicities. Eighty per cent of physicians reported giving pre-emptive hydration to patients prior to each dose of AmBd; however, in clinical practice this was only delivered 40% of the time.

In keeping with other studies, we found a high prevalence of AmBd-related toxicities; however, only 24% of these toxicities were classified as severe or life-threatening [3, 17, 25]. Half of the patients developed nephrotoxicity; two-thirds developed hypokalemia; and half developed anemia. The nephrotoxicity reported in this study was in keeping with experiences from elsewhere: this usually occurred in the second week of AmBd treatment and was mostly reversible if diagnosed and treated appropriately. We report a much higher prevalence of hypokalemia in this observational study than has been reported in clinical trial settings, where electrolyte supplementation was standardized [16]. Only 40% of interviewed physicians reported routinely administering KCl and in practice, very few patients actually received this supplementation (19% oral and 14% IV). Physicians may be reluctant to use KCl supplementation for fear of hyperkalemia and its complications. However, the high prevalence of hypokalemia during AmBd treatment in this and other studies suggests that this concern is unfounded, especially if laboratory tests are ordered as recommended [16, 18, 19]. Anemia is a common finding among HIV-infected hospitalized patients and a common toxicity associated with AmBd therapy [16, 26]. Of the patients with optimal Hb monitoring, a large proportion (54%) developed anemia during AmBd treatment. Severe, potentially life-threatening anemia, which requires urgent blood transfusion, was fortunately rare.

Despite adequate knowledge of the disease management guidelines by interviewed physicians, actual implementation of recommendations for preventing and monitoring for AmBd toxicities was particularly poor. Evidence-based guidelines for patient management are useful documents but will only impact on patient outcome if physicians are aware of them and if all members of the clinical team adhere to them. Although local guidelines to reduce AmBd-related toxicities have been developed, targeted training may be required to educate hospital-based pharmacists, nurses and doctors. It may also be useful for hospital pharmacists to attach step-by-step prevention and monitoring recommendations to the prescription chart when AmBd is released by the hospital pharmacy.

Use of liposomal amphotericin B in South Africa is currently prohibitive due to the high cost (approx. US$160 per 50 mg vial vs. approx. US$2.55 per 50 mg vial for AmBd). Liposomal amphotericin B is currently available through Gilead’s Visceral Leishmaniasis Treatment Expansion Program at a price of approx. US$16 per 50 mg vial. If a reduced price could also be negotiated for cryptococcosis in resource-limited settings, liposomal amphotericin B could potentially be used as a second-line agent in the subset of patients (approximately 25%) that develop AmBd-related nephrotoxicity. Liposomal amphotericin B is a much safer agent with fewer toxicities and use of this agent would eliminate the need for an intensive toxicity prevention and monitoring regimen [27, 28].

There were limitations in determining the presence of toxicities in this observational study, as laboratory testing was not performed according to a schedule. Many patients did not have appropriate tests ordered, even though all the hospitals in the study had access to laboratory facilities. Therefore, without optimal monitoring, we could not determine the impact that these toxicities had on patient outcomes as it is likely that patients who developed clinical signs of toxicities were more likely to be tested. We did not collect data on whether serial therapeutic lumbar punctures, which benefit patients with raised intracranial pressure and may be indicators of better patient care, were performed [29]. Our data showed that patients who had an initial intracranial CSF opening pressure measurement had better outcomes. Although this was not significant, it may indicate better patient care.

A limitation of conducting separate physician interviews was that those interviewed may not have been the same physicians treating the patients in the study. However, all physicians reported being directly involved in day-to-day management of patients with cryptococcosis. Other reasons could include recall bias, physicians’ orders not being directly followed or various institutional barriers to best practice, e.g. pharmacy stock outs.

A strength of this observational study was the large geographic area that it covered, as the ES sites, where the clinical data were collected, treat on average a quarter of the patients diagnosed with cryptococcosis in the country [12]. The aggregated results mask the excellent care provided at some hospital settings as well as the poor management of patients at others.

Although prevention or early diagnosis of cryptococcal disease among HIV-infected patients should be the primary focus from a public health perspective, our data show that once patients are admitted to hospital with cryptococcosis, there are still many areas of management that could be improved upon. As AmBd forms the backbone of an induction-phase regimen for CM, AmBd-related toxicities need to be prevented and properly managed. This study highlights a need for adherence to AmBd toxicity prevention, monitoring and management recommendations, as toxicities occurred frequently but are potentially preventable with adequate monitoring and supplemental therapies.

## Supporting Information

S1 Checklist STROBE Checklist of items that should be included in reports of cross-sectional studies: PNTD-D-16-00494R1: Prevalence and hospital management of amphotericin B deoxycholate-related toxicities during treatment of HIV-associated cryptococcal meningitis in South Africa. (DOC)

S1 Table Grading of toxicities among patients treated with amphotericin B deoxycholate and optimally monitored* during treatment: a comparison between those who had existing toxicity at baseline and those who developed new onset toxicity during treatment. Footnote: *Optimal monitoring: hemoglobin measured weekly whilst on amphotericin B deoxycholate (AmBd) therapy; or K or Cr measured biweekly whilst on AmBd therapy.
(DOCX)

S2 Table Comparison of adherence to recommendations for prevention and monitoring of amphotericin B deoxycholate-related toxicities at academic versus non-academic hospitals (n = 524). (DOCX)
