# Supplementary material for: Prevalence and Hospital Management of Amphotericin B Deoxycholate-Related Toxicities during Treatment of HIV-Associated Cryptococcal Meningitis in South Africa
Source: PLoS Negl Trop Dis. 2016 Jul 28;10(7):e0004865. doi: 10.1371/journal.pntd.0004865 (PMC4965057; doi:10.1371/journal.pntd.0004865)
Supplement: S1 Table — Footnote: *Optimal monitoring: hemoglobin measured weekly whilst on amphotericin B deoxycholate (AmBd) therapy; or K or Cr measured biweekly whilst on AmBd therapy. (DOCX) [file pntd.0004865.s002.docx]

| **Toxicity** | **Grading of toxicities (Division of AIDS)** | | | | | | | |  |
| --- | --- | --- | --- | --- | --- | --- | --- | --- | --- |
|  | **Grade 1 (Mild)** | | **Grade 2 (Moderate)** | | **Grade 3**  **(Severe)** | | **Grade 4 (Potentially life-threatening)** | |  |
|  | **N** | **%** | **N** | **%** | **N** | **%** | **N** | **%** | **p-value** |
| Nephrotoxicity |  |  |  |  |  |  |  |  |  |
| -present at baseline | 0 | 0 | 5 | 36 | 8 | 57 | 1 | 7 | 0.03 |
| -new presentation | 33 | 29 | 46 | 41 | 28 | 25 | 6 | 5 |  |
| Hypokalemia |  |  |  |  |  |  |  |  |  |
| -present at baseline | 12 | 24 | 24 | 48 | 9 | 18 | 5 | 10 | 0.09 |
| -new presentation | 43 | 37 | 46 | 39 | 25 | 21 | 3 | 3 |  |
| Anemia |  |  |  |  |  |  |  |  |  |
| -present at baseline | 10 | 31 | 7 | 22 | 7 | 22 | 8 | 25 | 0.03 |
| -new presentation | 27 | 60 | 10 | 22 | 3 | 7 | 5 | 11 |  |

Table S1: Grading of toxicities among patients treated with amphotericin B deoxycholate and optimally monitored* during treatment: a comparison between those who had existing toxicity at baseline and those who developed new onset toxicity during treatment.

*Optimal monitoring: hemoglobin measured weekly whilst on amphotericin B deoxycholate (AmBd) therapy; or K or Cr measured biweekly whilst on AmBd therapy.
