# Supplementary material for: Prevalence and Hospital Management of Amphotericin B Deoxycholate-Related Toxicities during Treatment of HIV-Associated Cryptococcal Meningitis in South Africa
Source: PLoS Negl Trop Dis. 2016 Jul 28;10(7):e0004865. doi: 10.1371/journal.pntd.0004865 (PMC4965057; doi:10.1371/journal.pntd.0004865)
Supplement: S2 Table — (DOCX) [file pntd.0004865.s003.docx]

Table S2: Comparison of adherence to recommendations for prevention and monitoring of amphotericin B deoxycholate-related toxicities at academic versus non-academic hospitals (n=524)

| **Parameter** | **Academic hospital (n=286)** | | **Non-academic hospital (n=238)** | |  |
| --- | --- | --- | --- | --- | --- |
|  | **n** | **%** | **n** | **%** | **p-value** |
| **Preventive parameters:** |  |  |  |  |  |
| Baseline blood tests | 192 | 67 | 141 | 59 | 0.062 |
| Pre-emptive hydration given | 110 | 38 | 101 | 42 | 0.356 |
| Intravenous potassium chloride given | 57 | 20 | 15 | 6 | <0.001 |
| Oral potassium chloride given | 53 | 19 | 48 | 20 | 0.636 |
| **Monitoring parameters:** |  |  |  |  |  |
| Daily fluid input/output monitoring | 273 | 98 | 179 | 76 | <0.001 |
| Hemoglobin | 95 | 33 | 47 | 20 | 0.001 |
| Potassium (serum) | 176 | 62 | 59 | 25 | <0.001 |
| Creatinine (serum) | 171 | 60 | 61 | 26 | <0.001 |

Optimal adherence to baseline blood test recommendations: hemoglobin, serum potassium and serum creatinine checked in the week preceding amphotericin B deoxycholate (AmBd) therapy

Pre-emptive hydration: 1L 0.9% saline administered prior to each dose of AmBd (optimal = pre-emptive hydration given for >80% of doses)

Intravenous potassium chloride (KCl): 1 ampoule (20 mEq) of KCl added to 1L normal saline prior to each dose of AmBd (optimal= given for >60% of doses)

Oral KCl: any tablets containing KCl administered daily whilst on AmBd (optimal= given for >60% of doses of AmBd)

Optimal hemoglobin monitoring: hemoglobin measured weekly whilst on AmBd therapy

Optimal potassium (K) or creatinine (Cr) monitoring: K or Cr measured biweekly whilst on AmBd therapy
